# A practical application of text mining to literature on cognitive rehabilitation and enhancement through neurostimulation

**DOI:** 10.3389/fnsys.2014.00182

**Published:** 2014-09-26

**Authors:** Puiu F. Balan, Annelies Gerits, Wim Vanduffel

**Affiliations:** Laboratory for Neuro-and Psychophysiology, Katholieke Universiteit Leuven Medical SchoolLeuven, Belgium; Athinoula A. Martinos Center for Biomedical Imaging, Massachusetts General HospitalCharlestown, MA, USA; Department of Radiology, Harvard Medical SchoolCharlestown, MA, USA

**Keywords:** text mining, transcranial magnetic stimulation, cognitive, rehabilitation, enhancement

## Abstract

The exponential growth in publications represents a major challenge for researchers. Many scientific domains, including neuroscience, are not yet fully engaged in exploiting large bodies of publications. In this paper, we promote the idea to partially automate the processing of scientific documents, specifically using text mining (TM), to efficiently review big corpora of publications. The “cognitive advantage” given by TM is mainly related to the automatic extraction of relevant trends from corpora of literature, otherwise impossible to analyze in short periods of time. Specifically, the benefits of TM are increased speed, quality and reproducibility of text processing, boosted by rapid updates of the results. First, we selected a set of TM-tools that allow user-friendly approaches of the scientific literature, and which could serve as a guide for researchers willing to incorporate TM in their work. Second, we used these TM-tools to obtain basic insights into the relevant literature on cognitive rehabilitation (CR) and cognitive enhancement (CE) using transcranial magnetic stimulation (TMS). TM readily extracted the diversity of TMS applications in CR and CE from vast corpora of publications, automatically retrieving trends already described in published reviews. TMS emerged as one of the important non-invasive tools that can both improve cognitive and motor functions in numerous neurological diseases and induce modulations/enhancements of many fundamental brain functions. TM also revealed trends in big corpora of publications by extracting occurrence frequency and relationships of particular subtopics. Moreover, we showed that CR and CE share research topics, both aiming to increase the brain's capacity to process information, thus supporting their integration in a larger perspective. Methodologically, despite limitations of a simple user-friendly approach, TM served well the reviewing process.

## Introduction

Gathering accurate and reliable information from web repositories became increasingly complex because of the exponential growth in the number of publications. For example, a PubMed search retrieved 9407 papers including 1172 reviews for TMS in “All Fields,” and the ratio became 8186/988 when the filter “[Title/Abstract]” was applied. Reading without some selection criteria becomes challenging. Even when selectively focusing on specific topics in a review, this increases the chances to miss trends shown only by huge bodies of literature. Thus, when processing vast corpora of publications, we are facing challenges that require automated solutions. One of the most promising approaches to alleviate these problems is to assist the human operator with computers running artificial intelligence applications. Here, we selected one of these applications, text mining (TM), and showed that TM will enable us to efficiently deal with huge amounts of information from the TMS-related literature.

Our approach was also motivated by the fact that, neuroscience has to make efforts to integrate data mining and TM when dealing with huge and diverse experimental datasets (Akil et al., 2011) and text documents. TM is able to catch the complexity of all relevant studies in an efficient manner. Statistical and natural language processing (NLP) procedures to “mine” the literature have been developed to address big data general problems (Dias et al., 2011). Here, we used a practical approach to promote TM as a tool for the reviewing process. Specifically, we selected a set of TM-tools that allowed user-friendly approaches to reveal relevant outcomes in large corpora of publications. Our intention was to use TM-tools that are not too demanding on programming skills, required knowledge and training period. Therefore, the example set of TM-tools could serve as an attractive guide map for researchers willing to incorporate TM in their work.

Next, we demonstrate the use of TM-tools in gaining basic insights into the relevant literature on cognitive rehabilitation (CR) and cognitive enhancement (CE) using transcranial magnetic stimulation (TMS). TMS is a valuable non-invasive perturbation method used to address fundamental and clinical neuroscience questions, both in human and animal models. Cognitive rehabilitation (CR-TMS) and cognitive enhancement (CE-TMS) are two important TMS applications. Indeed, TMS is establishing itself as major tool used in rehabilitation improving a wide variety of impaired mental functions (Miniussi and Rossini, 2011; Kammer and Spitzer, 2012; Vicario and Nitsche, 2013). Moreover, recent studies reported TMS-induced enhancements of normal brain functions (Brem et al., 2014; Luber and Lisanby, 2014).

The TM application to CR- and CE-TMS literature was focused on two main aspects. First, we aimed to show that TM could reveal the diversity of TMS applications in CR and CE, automatically retrieving trends already described in published reviews. Second, we specifically aimed to find trends only noticeable in big corpora of publications. The main feature of our findings is given by the statistical power of such analyses. Detailed analyses of the CR- and CE-TMS literature revealed relevant terms in the form of lists, topics and classes of terms associated with specific subtopics. It also showed relations between the relevant terms in the form of co-occurrences maps, groups of relevant terms with high probability co-occurrences and lists of relevant relational verbs. Moreover, the TM approach revealed conclusive sentences that appeared with a high probability. Finally, a large-scale corpora perspective showed that CR-TMS and CE-TMS share research topics allowing us to make inferences about their similarities. Although they start from specific states of the brain (impaired for CR and normal for CE), both aim to increase the brain's capacity to process information and to optimize adaptation. This unitary perspective is supported by fields that use TMS in diagnostic (TMS-DIAG) or in clinical and fundamental research (TMS-RES), which show that TMS is effective for changing and studying normal and abnormal brain processes. Accordingly, CR-TMS and CE-TMS also share research topics with these fields, showing their appurtenance to a larger context, which integrates diagnostic, fundamental research and fMRI studies.

## Text mining as a method to partially automate the reviewing of big corpora of publications

Scholarly journals and data sources are increasingly available in electronic and Open Access form. Nonetheless, availability is not enough to extract specific information, mainly due to the abundance of information. TM comes with solutions for this problem offering automated methods to extract condensed information hidden within huge volumes of publications. TM can be achieved using several complementary approaches (Cohen and Hunter, 2008). Co-occurrence-based methods look for concepts that occur in the same unit of text (sentence/abstract) and posit a relationship between them. The statistical or machine learning systems rely on statistic properties of the text and work by building classifiers that operate on any level, from labeling part of speech to classifying full sentences or documents. The rule-based systems use knowledge about how language is structured and about how domain relevant things, facts and their relationships are stated in publications. The main areas/stages of TM are (Lourenco et al., 2009): Information Analysis that includes Information Retrieval and Information Extraction (e.g., Name Entity Recognition, Relationship Extraction, document classification and summarization); Information Synthesis that uses the databases generated by Information Extraction for answering simple questions, discovering new information and generating hypotheses.

A wide variety of publications and software tools approach TM at different levels of complexity, hampering the selection of relevant TM-tools. We chose a specific set of TM-tools, aiming to evaluate how they can help the TM-non-specialist accelerate the review of big corpora of literature, using the following criteria:

- Allow a user-friendly approach by selecting TM-tools requiring medium investments in training, programming and specific knowledge.
- Use free open-source software, selected based on features like: easiness of installation; quality of the documentation and support; accessible formats for input and output.
- Use TM-tools with general functionalities like: allowance of document corpora; pre-processing the text; built-in biomedical Name Entity Recognition; queries to a document or corpora; support for ontologies and terminologies.

We used three groups of TM-tools that served different purposes:

- Basic resources that gave foundation to TM: the MeSH browser; the PubMed repository of publications; repositories of NLP resources (e.g., Neuroscience Information Framework, National Institute of Neurological Disorders and Stroke).
- TM-tools II (Table 1) that are web-based ready-to-use tools requiring no programming efforts and performing simple TM tasks (Lu, 2011).
- TM-tools III that were used in the final stage of the study optimized for the reviewed topics:
  1. Statistical or machine-learning-based approaches: Mallet (McCallum, 2002), Text to Matrix Generator (TMG) (Zeimpekis and Gallopoulos, 2006) and Matlab applications for NLP.
  2. TM-tools with predefined NLP processing stream: KH Coder (Higuchi, 2012).
  3. Integrated environments for visual programming of NLP: VisualText (Meyers, 2003; Alfred et al., 2014).
  4. Biomedical TM rule-based approaches using fully automated stages in text processing: Anote2 (Lourenco et al., 2009) and Biological Research Assistant for TM (BioRAT) (Corney et al., 2004).

**Table 1:**
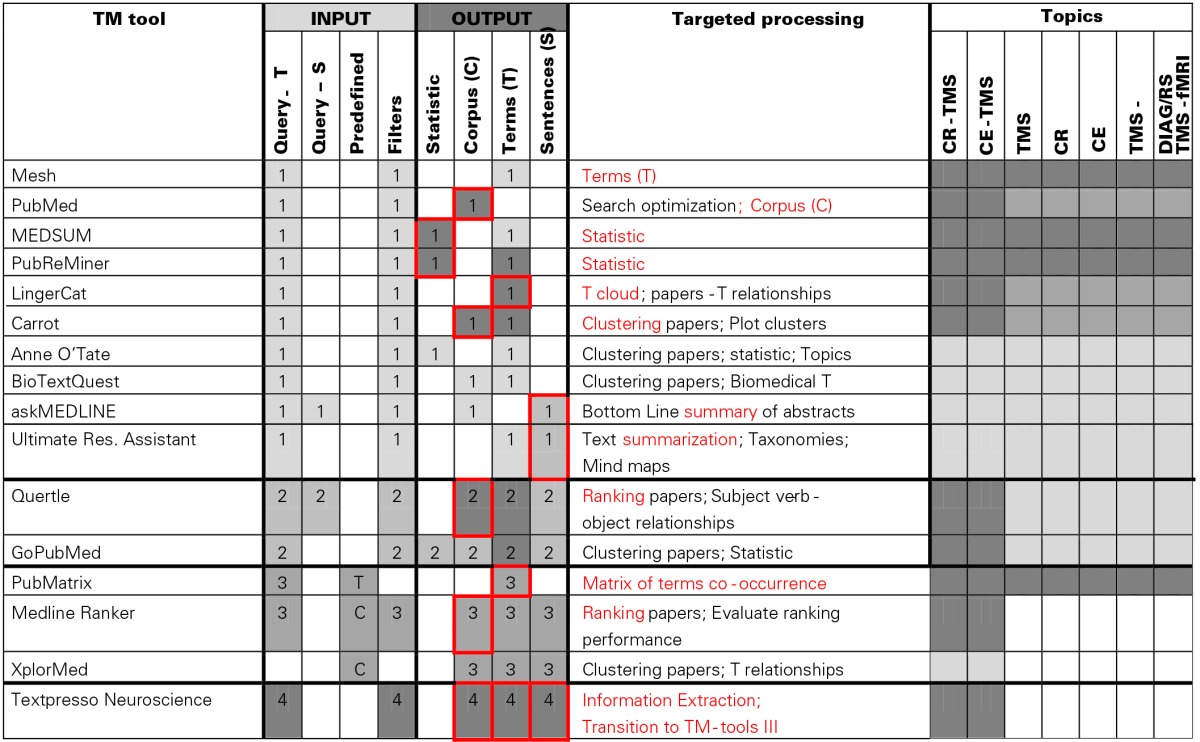
**TM-tools II presented in the order (indicated by numbers) of their use**.

*Gray scale qualitatively codes their weights in TM (the darker, the higher the importance). Each subgroup (1–4) includes kernels of TM-tools (circled red) and “targeted processing” (red text) that have the highest importance for our study. INPUT: query terms (T) = query using combinations of terms; query sentences (S) − query using free text or questions; predefined = fixed user-predefined input consisting of lists of terms or publications; filters = tuning the search using supplementary terms, constrains regarding the type/part of the publication to be processed etc. OUTPUT: Statistic about authors, journals, papers per year, topics; corpus (C) = retrieving sets of relevant papers; terms (T) = extracting lists of frequent and relevant terms and their relationships; sentences (S) = extracting relevant sentences and paper summaries. Targeted processing = processing from the TM-tool repertoire used for this review. The last 7 columns show topics whose corpora of publications were studied with TM (gray scale codes the weight of the approach). References: MEDSUM (Bridges-Webb, 1986), PubReMiner (Koster, 2008), LingerCat (Sarkar et al., 2009), Carrot2 (Carpineto et al., 2009), Anne O'Tate (Smalheiser et al., 2008), BioTextQuest (Iliopoulos et al., 2001), askMEDLINE (Fontelo et al., 2005), Ultimate Research Assistant (Hoskinson, 2005), Quertle (Giglia, 2011), GoPubMed (Doms and Schroeder, 2005), PubMatrix (Becker et al., 2003), Medline Ranker (Fontaine et al., 2009), XplorMed (Perez-Iratxeta et al., 2001), Textpresso for Neuroscience (Muller et al., 2008)*.

## A practical application of the text mining to literature on cognitive rehabilitation and enhancement through neurostimulation

To show that TM enables us to efficiently deal with big corpora of publications and for publishing practical reasons, the TM application to CR- and CE-TMS literature was limited to few aspects. First, we aimed to show that TM could retrieve the diversity of TMS applications in vast corpora of publications about CR and CE (see Cognitive Rehabilitation and Enhancement Accomplished with TMS), automatically retrieving trends already described in published reviews (see Discussions and Conclusions). Second, we looked for trends noticeable only in big corpora of publications, relying on the statistical power of the analyses and including results like topics' occurrence frequency and relationships, relevant relational verbs, and high probability conclusive sentences (see Cognitive Rehabilitation and Enhancement Accomplished with TMS). Furthermore, we analyzed large context relationships between topics showing how CR- and CE-TMS integrate with diagnostic, fundamental research and fMRI studies (see A General Context).

Using TM to efficiently review corpora of publications requires roughly three stages: pre-TM-processing, TM-processing, and post-TM-processing. In this paper, we focused on the TM-processing by showing mainly the “raw” TM results. Accordingly, the seemingly redundant diversity of results is determined by our intention to illustrate few similar results given by different TM-tools.

We used a multi-stage and multi-tool approach ordered by the complexity in TM, which was gradually increased, starting with TM-tools II and continuing with TM-tools III. The same analysis was performed with few TM-tools (Table 2), which can be regarded as alternative solutions for the same problem. This helped us to cope with the limited perspective offered by different TM-tools, to perform comparisons and cross-validations, and to build synthetic results.

**Table 2:** **Number of publications retrieved from PubMed using search queries defined previously**.

**Search query**	**Publications per search filter:**		
	**TIAB**	**PBRK**	**TIABREV**
**CR-TMS**	**4074**	**0.913**	**641**
**CE-TMS**	**181**	**0.956**	**41**
TMS	8386	0.909	1018
CR	4220	0.896	914
CE	1235	0.895	312
TMS-DIAG	212	0.994	68
TMS-RES	360	0.994	93
TMS-fMRI	875	0.906	211

*PBRK, the probability of correct ranking of a random positive-negative pair of publications determined with Medline Ranker*.

The main classes of TM processing on the selected corpora were:

– Statistics about the number of publications, authors, journals; thematic/MeSH headings division of the field; clustering of publications; selection and ranking of relevant corpora of papers necessary for TM-tools III (all performed with TM-tools II).
– Extract relevant terms and their relationships, involving: categorization of key concepts; building correlation matrices for relevant terms and showing the most informative correlations; showing the topological maps of the main terms, using their relationships inferred from co-occurrence in the same text units.
– Retrieve automatically relevant sentences, study their probability of occurrence, and identify parts-of-sentence (e.g., predicates) relevant for deriving conclusions.
– Build a “map of science,” which characterize large-scale relationships between multiple topics. Each topic-topic relationship was evaluated based on common relevant terms retrieved from each corpus, similar thematic clustering of the publications, common authors, journals and publications approaching both topics.

**Figure 1:**
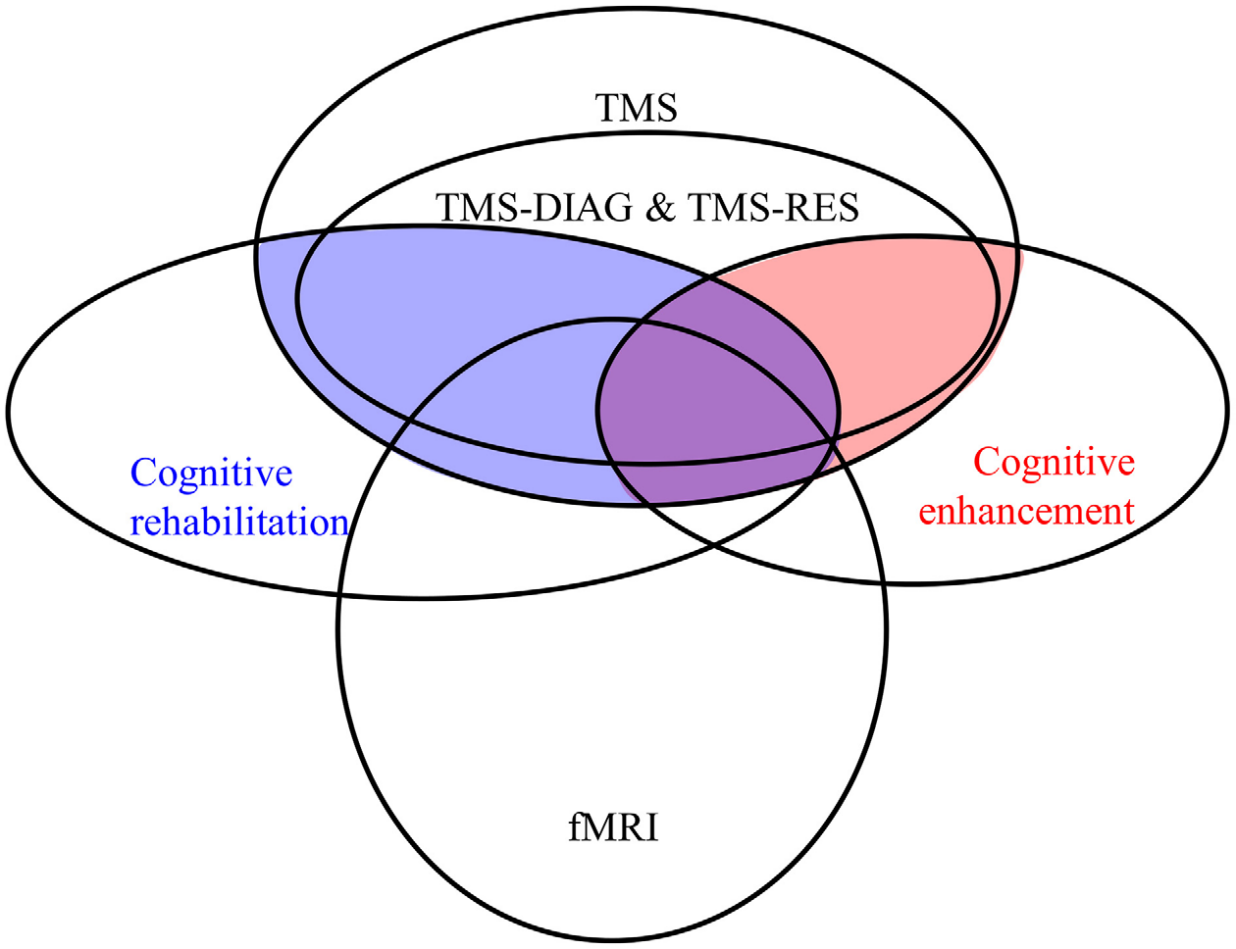
**Qualitative approximation of a larger context for CR- and CE-TMS**. Topics are represented by ellipses with CR-TMS (filled blue) and CE-TMS (red) representing intersections of topics.

To create a larger context allowing a better understanding of CR- and CE-TMS, we selected topics like TMS, CR, CE, TMS-RES, TMS-DIAG, and TMS-fMRI. Figure 1 presents a “qualitative hypothesis” about the topology of this context and we used TM to test it. Corpora of publications for each topic were retrieved using the following PubMed queries:

– For CR-TMS: (“transcranial magnetic stimulation”) AND (“cognitive rehabilitation” OR “rehabilitation” OR “cognitive therapy” OR “therapy” OR “cognitive recovery” OR “recovery” OR “cognitive treatment” OR “treatment” OR “cognitive repair” OR “neurorehabilitation” OR “improvement” OR “decrease”).
– For CE-TMS: (“transcranial magnetic stimulation”) AND [(“cognitive enhancement”) OR (“cognitive augmentation”) OR (“cognitive improvement”) OR (“cognitive enrichment”) OR (“cognitive amelioration”) OR (“neuroenhancement”)].
– For TMS: “transcranial magnetic stimulation.”
– Queries for CR/CE include the second operand of the AND operator in the CR-TMS/CE-TMS queries.

All queries were used separately with two filters (“[Title/Abstract]” (TIAB-filter) and “[Title/Abstract] AND Review” TIABREV-filter) creating two separate corpora of abstracts: TIAB-corpora (the main target for TM) and TIABREV-corpora (used for comparisons). Empty or less relevant abstracts were removed from the corpora. Corpora were also compared with local databases and missing publications were added manually.

Finally, the TM results were evaluated in few ways. First of all, we used TM-tools that are already tested and evaluated, building our results on this general basis. Second, we used the *post-hoc* judgment of the system outputs (Cohen and Hunter, 2008) in few stages, and compared: results of similar processing (e.g., term extraction) performed with different TM-tools using TIAB-corpora (see A Practical Application of the Text Mining to Literature on Cognitive Rehabilitation and Enhancement Through Neurostimulation); results of similar processing using TIAB-corpora vs. TIABREV-corpora (see A Practical Application of the Text Mining to Literature on Cognitive Rehabilitation and Enhancement Through Neurostimulation); TM-results vs. manual-curated results (see Discussions and Conclusions).

### A general context

We used TM-tools II to determine relationships that put CR- and CE-TMS topics in the same neighborhood on a “map of science.” An outline of the main results includes:

1. MeSH headings mention TMS as a preferred term, defined by its use in brain mapping, neurophysiology and treatment of depression (replacing electroconvulsive therapy), while paired-pulse TMS, rTMS and single-pulse TMS are narrower concepts. The MeSH tree considers that TMS belongs to the fields of neurological diagnostic and therapeutic techniques. CR is related with MeSH headings like Cognitive Therapy, Rehabilitation and Treatment/Rehabilitation Outcome, and CE is related with Cognitive Therapy, Nootropic Agents and Cognitive Enhancers.
2. The number of the publications retrieved from PubMed (Table 2) for each topic.

It is noteworthy that a large number of reviews were written for each topic.

**Figure 2:**
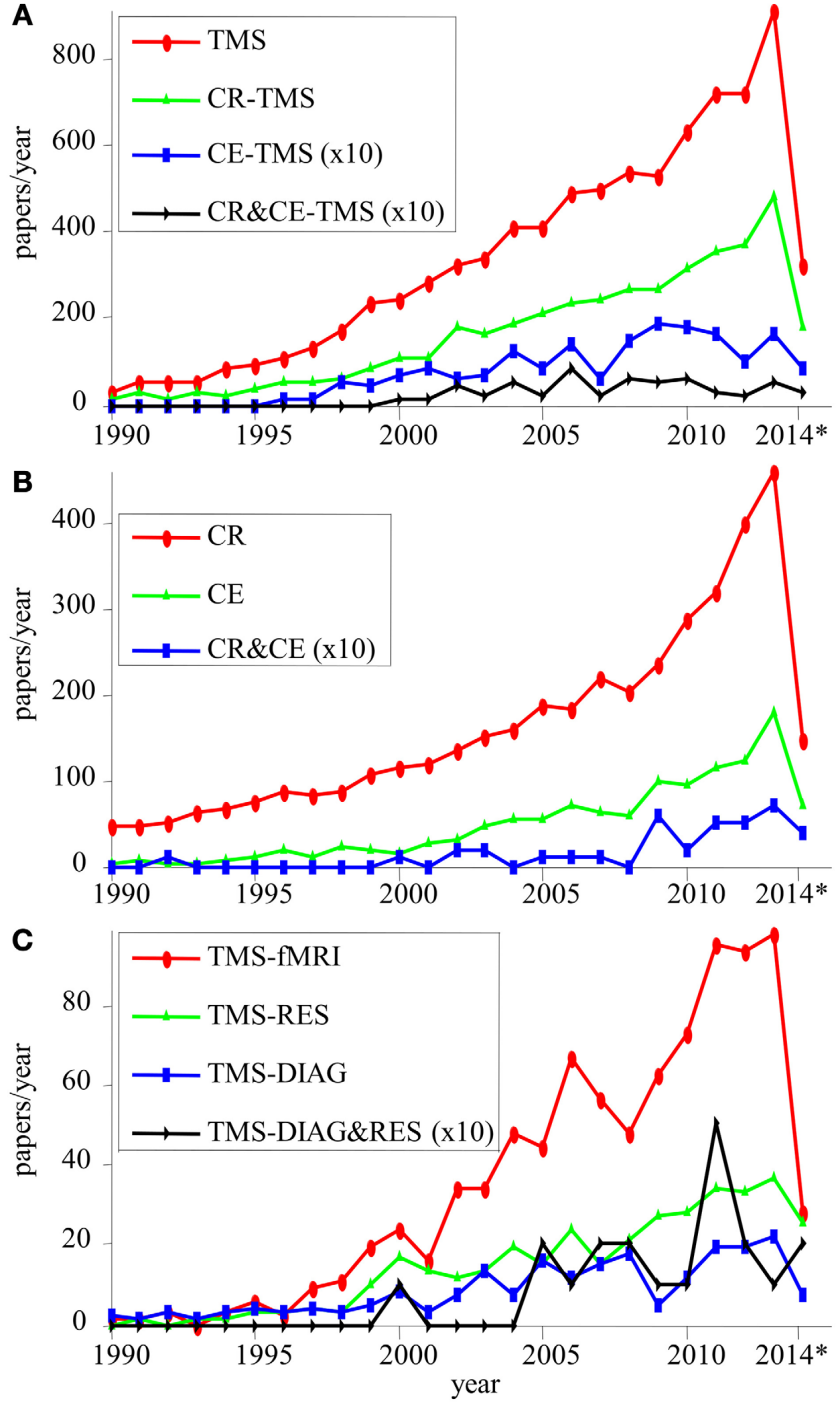
Number of publication per year (2014^*^, only the first 3 months) for different topics: TMS, CR-TMS, and CE-TMS (A); CE and CR (B); applications of TMS in research (TMS-RES), diagnostic (TMS-DIAG) and concurrent TMS-fMRI (C). Results for topics represented by small numbers of publications are multiplied by 10 (×10). We show also results for conjunctions of topics: (CR&CE-TMS) **(A)**; (CR&CE) **(B)**; (TMS-DIAG&RES) **(C)**.

**Figure 3:**
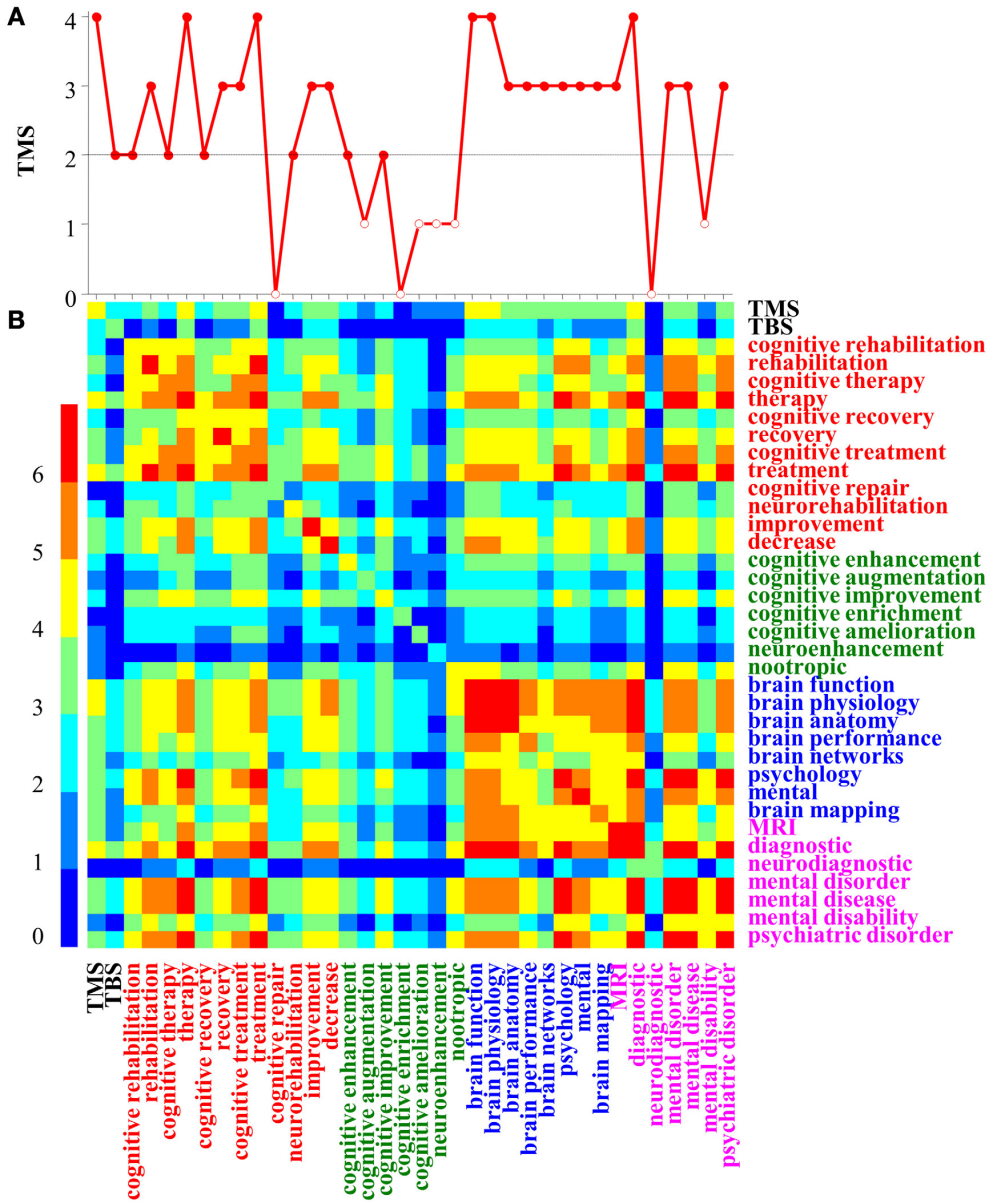
**Co-occurrence matrix built with PubMatrix**. The matrix **(B)** represents the decimal logarithm of the number of publications retrieved from PubMed using queries combining all possible conjunctions of pairs of terms (e.g., TMS and neurorehabilitation), which label the lines and the rows of the matrix. Panel **(A)** represents the first line in the matrix, showing co-occurrences involving the term TMS. Different colors (see the left color-coding bar) represent different powers of 10. The color of the text marks terms associated dominantly with: TMS (black); CR-TMS (red); CE-TMS (green); TMS-RES (blue); TMS-DIAG (purple).

3. The number of publications per year evaluating the interest for each topic (Figure 2), which showed the following trends:
  – The interest for all topics increased in the last decades;
  – TMS generated numerous publications per year;
  – CR-TMS has a stronger representation than CE-TMS;
  – Across-topics perspectives (CR & CE; CR & CE-TMS; TMS-DIAG & RES) are less represented.
  – Fundamental research (e.g., TMS-RES, TMS-DIAG) is less represented than practical applications (e.g., CR-TMS).
4. Co-occurrence matrix for terms used to build the search queries, retrieved with PubMatrix (Figures 3A,B). We made the following observations:
  – TMS co-occurred very frequently (publications ~10^3^) with terms like therapy, treatment, brain function, brain physiology, and diagnostic (mainly CR-TMS);
  – TMS co-occurred frequently (publications ~10^2^) with rehabilitation, recovery, cognitive treatment, improvement, decrease, brain anatomy, brain performance, brain networks, psychology, brain mapping, MRI, mental disorder, mental disease and psychiatric disorder (mainly CR-TMS and CE-TMS);
5. Frequent terms co-occurring in different corpora (included between brackets):
  - Among the first 10 MeSH headings, we mention:
    – [CR-TMS]: Motor Cortex/physiology, TMS/methods, Evoked Potentials, Treatment Outcome, Evoked Potentials, Electromyography, Functional Laterality, Stroke, Brain, Motor, (Major) Depressive Disorder;
    – [CE-TMS]: TMS/methods, Brain Mapping, Brain, Functional Laterality, Cognition, Psychomotor Performance, Reaction Time/physiology, Motor Cortex/physiology, Cognition Disorders, Prefrontal Cortex/physiology.
  - Among the first 100 common MeSH headings (average frequency range [51, 2010]) for groups of topics (included between brackets), we mention:
    – [CR-TMS; CE-TMS; TMS; TMS-fMRI]: Brain, Cognition, Brain Mapping, Electromyography, Evoked Potentials, Motor Skills, MRI, Motor Cortex/physiology, Neural Inhibition, Neuronal Plasticity, Neuropsychological Tests, Parietal Lobe, Prefrontal Cortex, Psychomotor Performance, Reaction Time, TMS/methods, Treatment Outcome;
    – [CR-TMS; CR]: Brain, Chronic Disease, Cognition, (Major) Depressive Disorder, MRI, Motor Cortex, Neuropsychological Tests, Psychomotor Performance, Recovery Of Function, Schizophrenia, Severity Of Illness Index, Treatment Outcome, Neuronal Plasticity/physiology, Recovery of Function/physiology, Stroke;
    – [CE-TMS; CE]: Attention, Brain, Cognition, Learning, MRI, Memory, Neurons, Neuropsychological Tests, Psychomotor Performance, Reaction Time, Prefrontal Cortex, Treatment Outcome, Cognition Disorders/diagnosis and etiology.
  - Other trends noticed using the TM-tools II:
    – Different groups of topics (e.g., [CR-TMS; CE-TMS]; [TMS; TMS-fMRI; TMS-RES; TMS-DIAG]) are connected as shown by common headings/terms;
    – CR-TMS related terms: motor cortex, treatment, excitability, facilitation, antidepressant, plasticity, MEP, stroke, Parkinson, severity of illness index, treatment outcome, (major) depressive disorder, schizophrenia, stroke/complications, neuronal plasticity, rehabilitation, cognition, recovery, parietal lobe, prefrontal cortex, psychomotor performance;
    – CE-TMS related terms: prefrontal cortex, DLPFC, cognition, psychomotor performance, attention, learning, memory, performance, placebo, cognition disorders, severity of illness index, treatment outcome, neuroenhancement, Hebbian, neurofeedback, enhancement, improvement;
    – [CR-TMS; CE-TMS] and [CR; CE] groups of topics have “mental diseases” and “treatments” as common subtopics, thus supporting the hypothesis that the relative improvement of the mental performance is a common aspect of their definitions.
6. The first 5 authors for different topics (all including Pascual-Leone A on the first place):
  – [CR-TMS]: Fregni F, Daskalakis ZJ, Fitzgerald PB, Rothwell JC;
  – [CE-TMS]: Walsh V, Fregni F, Cowey A, Miniussi C;
  – [TMS]: Rothwell JC, Hallett M, Cohen LG, Fitzgerald PB;

Some authors covered several of the selected topics (e.g., common authors for [CR-TMS; CE-TMS; TMS; TMS-fMRI]: Pascual-Leone A, Cohen LG, Fregni F, Lisanby SH, Miniussi C, Rothwell JC), and this is an indirect argument for potential connections between topics.

**Figure 4:**
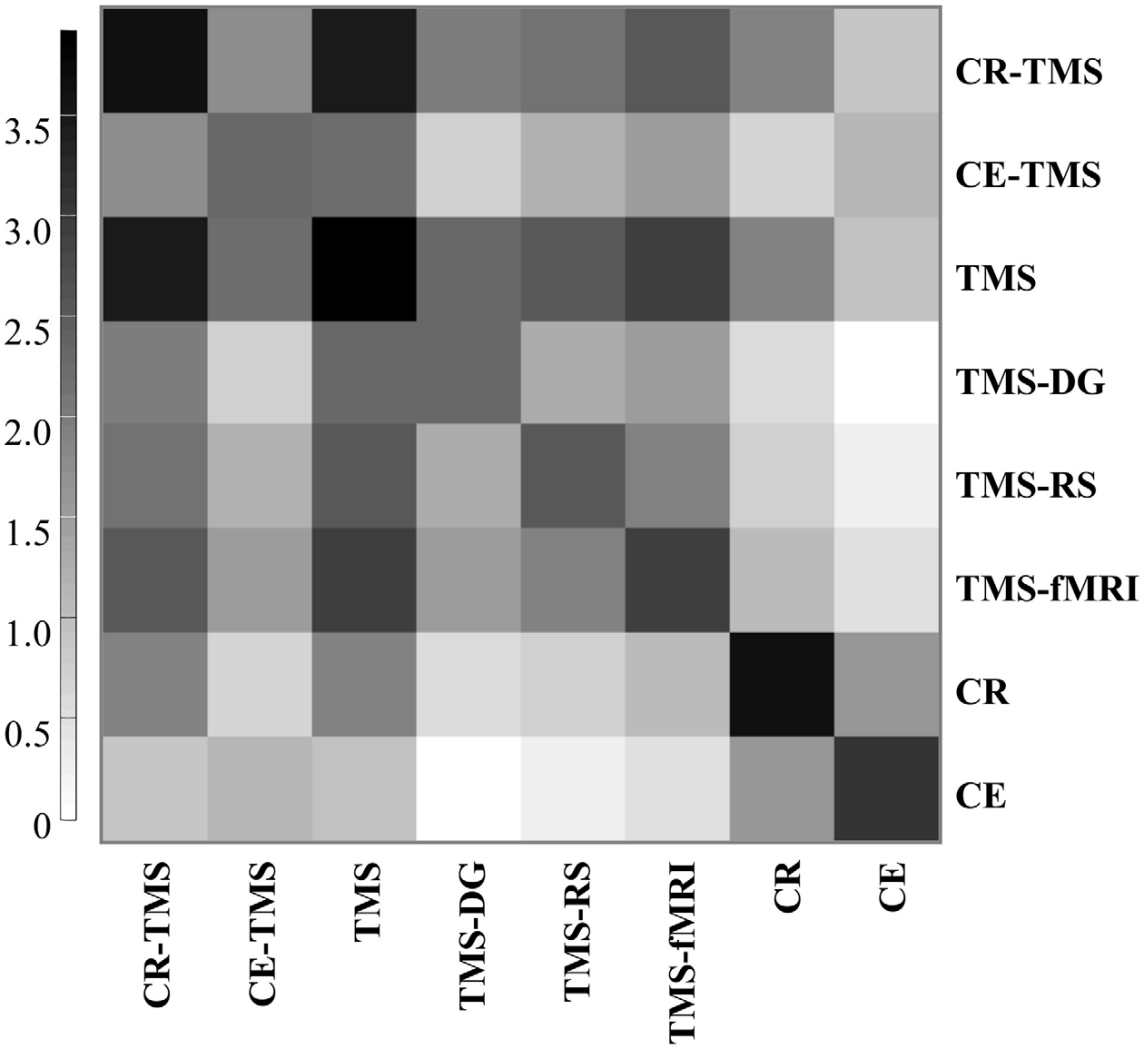
**Number of common publications (in decimal logarithmic scale) for pairs of corpora representing topics labeling the rows and the columns of the matrix**. Different shades of gray (see the color-coding bar) represent different powers of 10.

7. Finally, comparisons between corpora for different topics showed relevant numbers of common publications (Figure 4), thus emphasizing a unitary context. Consistently, clustering the publications for each topic using Carrot2 showed also different clusters sharing publications.

### Cognitive rehabilitation and enhancement accomplished with TMS

First, we need to reiterate that the relevance of all the retrieved terms is based on the idea that words co-occurring frequently in the abstracts are related in ways intrinsically constrained by the topic of the abstracts. Moreover, our specific selection of publications guarantees that term like TMS (all protocols) are present in all abstracts included in corpora. Thus, TMS is strongly related with all frequent terms retrieved with different TM-tools. Specifically, if, for example, the frequent terms are treatment and depression, this means that TMS is used frequently in depression treatment.

We used TM-tools III to increase our insights in CR-TMS and CE-TMS literature, and selected the following groups of results:

1. Statistical or machine-learning-based NLP:
  a. Topic modeling with Mallet (500 iterations, 7 topics, topics proportion threshold 0.05, and removed the standard Mallet stop-words). Each topic is a set of terms used with high probability by the authors, thus reflecting a specific thinking pattern involving TMS. Without knowing anything about the meaning of the words in a text, topic modeling assumes that any piece of text is written by selecting words from possible topics. Thus, it becomes possible to mathematically decompose a text into probable topics from which the words originated.

Example topics separated in CR/CE related terms (brackets) and TMS related terms (double brackets):

a1. CR-TMS:
  – [treatment, depression, clinical, therapy, disorder, major, antidepressant, electroconvulsive therapy], [transcranial, magnetic, stimulation, effective];
  – [motor, stroke, patients, recovery, function, hemisphere, affected, rehabilitation, limb], [magnetic, transcranial, stimulation].

The [CR] kernel of 2 topics includes CE-related terms (e.g., [cortex, learn, activity, visual, memory, performance, area]).

a2. CE-TMS:
  – [cortex, DLPFC, prefrontal, dorsolateral, memory, processing], [stimulation, applied, TMS, effect, frequency], significant;
  – [cognitive, brain, healthy, functions, enhance, neuroenhancement, parietal, cognition], [stimulation, magnetic, TMS], studies.

The [CE] kernel of 3 topics includes CR-related terms (e.g., [improvement, depression, treatment, deprivation, sleep, occipital, functional, disease, fluoxetine]).

b. Term-related queries with TMG toolbox. Document retrieval relies often on matching terms from documents with those from queries. However, natural languages present some challenges (e.g., polysemy, synonymy) that render term-matching inaccurate. TMG is using latent semantic analysis to overcome these problems (Landauer et al., 1998), based on the application of singular value decomposition of a term-by-document matrix (TDM).

We performed the indexing for each corpus creating new TDMs using “common-words” stop-list, logarithmic local weights, “GfIdf” global term weighs, normalization of terms, removing of alpha-numerics and numbers. For dimensionality reduction and TDM best rank approximation, we selected the dimensionality via the use of profile likelihood (Zhu and Ghodsi, 2006). Example results:

b1. For CR-TMS, the TDM included 607 documents, 6387 terms with average 111 indexing terms/document for the query matrix, and the best rank approximation 360. Among the best retrieved terms (apparition frequency > 86; range [870, 896] terms), we mention: [stimulation, rTMS, treatment, TMS, brain, motor, depression, patients, stroke, cortical, therapy, clinical, effects, disorders, pain, cortex] in the first 20; [recovery, disorder, schizophrenia, tinnitus, excitability, cognitive, plasticity, antidepressant, rehabilitation, mechanisms, psychiatric, symptoms, aphasia, language, noninvasive, sham, EEG, Parkinson, resistant, human, induced, movement, neglect] in the next 80; [cerebral, epilepsy, deep, migraine, neural, bipolar, visual, OCD, seizures, acute, neurological, diagnosis, auditory, neuroimaging, reorganization, prefrontal, inhibition, improvement, mood, neuropsychiatric, neuromodulation] in the next 50 terms.
b2. For CE-TMS, the TDM included 174 documents, 3235 terms with average 117 indexing terms/document for the query matrix, and the best rank approximation 174. Among the best retrieved terms (apparition frequency > 37; range [776, 1010] terms), we mention: [TMS, rTMS, motor, cortex, brain, visual, memory, cognitive, performance, parietal, healthy, patients, learning, improvement, enhancement] in the first 20; [excitability, neural, DLPFC, facilitation, plasticity, attention, control, prefrontal, network, evoked, neuroscience, perception, EEG, mechanisms, modulation, semantic, training, frontal, FEF, treatment, fluoxetine, neuroenhancement, sleep, emotional, skill, inhibition, Parkinson] in the next 80; [fMRI, MEP, Wernicke, search, discrimination, pain, cognition, increased, encoding, oscillatory, language, psychiatric, decrease, experimental, stroke, illness, major, effective, resistant, improve] in the next 50 terms.

2. Processing performed with KH Coder:
  a. Determine the co-occurrence network for CR-TMS and CE-TMS corpora using 7 sets of coding rules. A coding rule is a list of terms connected with OR (|), AND (&) or NOT used to “focus” the TM of the corpora toward specific topics. We used coding rules (marked by brackets) that were common for CR-TMS and CE-TMS: [TMS] (transcranial & magnetic & stimulation | rTMS | TBS etc.); [mental disabilities] (depression | stroke | schizophrenia etc.); [brain functions] (memory | learning | attention etc.); [research methods] (fMRI | EEG etc.); [verbs-] describing negative effects of the stimulation (fail | worsen | damage | debilitate | miss etc.). To these we added a set of rules specific to CR-TMS/CE-TMS: [verbs+] describing positive effects for CR-TMS (rehabilitate | treat | recover | restore etc.) or CE-TMS (enhance | improve | augment | strengthen etc.); [CR-TMS-effects] (rehabilitation | cognitive & rehabilitation | cognitive & therapy | neurorehabilitation etc.); [CE-TMS-effects] (performance | enhancement | neuroenhancement | neuromodulation etc.). The co-occurrence networks revealed that the terms from the coding rules [TMS], [CR-TMS-effects], [mental disabilities] and [Verbs+] have the highest co-occurrence frequency in the CR-TMS corpus. The CE-TMS corpus is characterized by high co-occurrence frequency of the terms from the coding rules [TMS], [CE-TMS-effects] and [Verbs+].
  b. Determine the keyword-in-context (KWIC) collection for terms in both CR-TMS (Table 3) and CE-TMS (Table 4). The KWIC statistic revealed information about both the relevant terms co-occurring with specific query terms (e.g., TMS) and statistical regularities (e.g., probable word positions) about the way the scientists build their statements.
3. Summary of results obtained with VisualText, ANote2, BioRAT and our Matlab applications (MAPP). All TM-tools used similar resources, including: dictionaries (mental disabilities, brain anatomy, cognitive processes, built using Neuroscience Information Framework (NIF) resources; CR-TMS-effects, CE-TMS-effects and TMS, built using our term statistic, similar with KH Coder coding rules); ontologies (NIF gross-anatomy and NIF dysfunction; human disease and neuro-behavior ontology from The Open Biological and Biomedical Ontologies Foundry); lexical words (Mallet stop-words; verbs+ and verbs- for CR-TMS and CE-TMS, similar with KH Coder rules). Each dictionary/ontology could be considered a class of terms (indicated by brackets; e.g., [mental disabilities]).

**Table 3:** **KWIC examples for CR-TMS**.

**Word**	**Total**	**LT**	**RT**	**L5**	**L4**	**L3**	**L2**	**L1**	**KW**	**R1**	**R2**	**R3**	**R4**	**R5**
Treatment	160	63	97	25	11	11	16	0	TMS	5	17	53	12	10
Depression	113	37	76	8	11	18	0	0	TMS	0	22	13	14	27
Alzheimer	100	55	45	20	13	19	3	0	TMS	2	12	10	5	16
Therapy	98	58	40	25	4	3	26	0	TMS	5	3	6	22	4
fMRI	79	57	22	14	6	19	18	0	TMS	0	3	8	0	11
Stroke	50	35	15	9	5	11	10	0	TMS	0	4	3	4	4
Disorder	38	27	11	9	3	2	13	0	TMS	0	0	6	4	1
Recovery	34	28	6	15	6	4	3	0	TMS	0	2	0	0	4
Schizophrenia	15	12	3	5	1	2	4	0	TMS	0	1	0	0	2
Parkinson	14	6	8	2	4	0	0	0	TMS	0	5	1	1	1
Improvement	13	5	8	3	0	1	1	0	TMS	0	0	2	1	5
Neurorehabilitation	20	14	6	7	0	1	6	0	TMS	0	2	1	1	2

*Columns show the number of co-occurrences of the context word (left) at different positions left/right (L5-L1/R1-R5) in the sentence relative to the query keyword (KW) and their total (LT/RT)*.

**Table 4:** **KWIC examples for CE-TMS**.

**Word**	**Total**	**LT**	**RT**	**L5**	**L4**	**L3**	**L2**	**L1**	**KW**	**R1**	**R2**	**R3**	**R4**	**R5**
Induce	29	6	23	2	1	3	0	0	TMS	3	15	1	2	2
Performance	24	12	12	5	4	3	0	0	TMS	0	1	7	2	2
EEG	18	5	13	2	0	1	2	0	TMS	0	11	1	1	0
Paired-pulse	17	16	1	0	1	2	0	13	TMS	0	0	0	0	1
Cognitive	16	4	12	2	0	2	0	0	TMS	1	2	6	2	1
Memory	13	9	4	2	3	3	1	0	TMS	0	1	1	2	0
Stimulation	13	5	8	1	2	0	2	0	TMS	2	0	3	2	1
fMRI	9	7	2	1	1	3	2	0	TMS	0	2	0	0	0
Enhance	8	3	5	1	1	1	0	0	TMS	1	2	0	2	0
Facilitate	7	1	6	1	0	0	0	0	TMS	1	1	0	3	1
Improve	6	1	5	0	0	1	0	0	TMS	1	3	0	0	1
Perception	5	3	2	0	2	1	0	0	TMS	0	0	0	0	2

The text processing relied on predefined streams of processing or on existing libraries of examples (e.g., TAIParse general text analyzer for VisualText). Our Matlab applications (MAPP) were used to handle the results, to perform relation extraction and to extract sentences with high probability of occurrence (low perplexity coefficients, PP).

We summarized here few results from the following processing: Evaluate term frequency and co-occurrences and perform Name Entity Recognition using lexical resources (ANote2, MAPP); Relations Extraction (ANote2, MAPP); Extract relevant sentences (VisualText, BioRAT, MAPP). The selected results are:

a. Terms statistic from Name Entity Recognition applied to CR-TMS (ANote2; 27506 annotations):
  – Top terms (number of occurrences): treatment (1177), rTMS (1000), brain (982), TMS (1410), therapy (503), depression (456), stroke (420);
  – Classes of terms (class per document, number of occurrences for all terms of the class): [neuro-behavior ontology] (22.8, 14903), [CR-TMS-effects] (4.8, 3163), [TMS] (4.3, 2811), [mental disabilities] (3.2, 2073), [cognitive processes] (1.6, 1028), [CE-TMS-effects] (0.2, 137);
  – Examples from the detailed class statistic (ANote2, MAPP; parentheses indicate number of occurrences):
    ° [TMS]: rTMS (1301), TMS (1466), TBS (32);
    ° [CR-TMS-effects]: treatment (1177), decrease (27), inhibition (96), antidepressant (175), antipsychotic (23), neurorehabilitation (45), recovery (286), rehabilitation (171), therapy (503);
    ° [mental disabilities]: ADHD (25), Alzheimer's (14), OCD (92), Parkinson (104), Tourette (9), anxiety (49), auditory hallucinations (24), bipolar depression (27), bipolar disorder (25), depression (462), epilepsy (77), major depressive disorder (111), neglect (68), psychiatric disorders (80), schizophrenia (158), seizures (69), stress (25), stroke (422), tinnitus (153);
b. Terms statistic from Name Entity Recognition applied to CE-TMS (ANote2; 7404 annotations):
  – Top terms (number of occurrences): TMS (727), rTMS (360), brain (207), cognitive (131), performance (130), facilitation (67);
  – Classes of terms (class per document, number of occurrences for all terms of the class): [neuro-behavior ontology] (22.97, 4111), [TMS] (6.23, 1116), [CE-TMS-effects] (3.70, 663), [cognitive processes] (3.49, 624), [mental disabilities] (0.35, 63), [CR-TMS-effects] (0.32, 57);
  – Examples from the detailed class statistic (ANote2, MAPP):
    ° [TMS]: TMS (739), rTMS (365);
    ° [CE-TMS-effects]: activation (28), cognitive (131), cognitive enhancement (19), enhancement (34), facilitation (67), improvement (29), neuroenhancement (27), performance (130), performance enhancement (8), rehabilitation (27), therapy (37);
    ° [cognitive processes]: attention (53), cognition (25), learning (70), memory (85), working memory (46), perception (37), visual search (19), skill acquisition (12), consolidation (9), decision (14), emotion (8), encoding (23), language (21), movement (17), recognition (13), semantic processing (2), speech (7);
c. Relationship Extraction for CR-TMS (ANote2; 27506 annotations). From 6754 relations, 2163 were verb associated relations, and we found among the top 30 the following verbs: induced, related, controlled, based, treating, underlying, compared, modulate, affected, applied, provide, discuss, to study, has been used, combined, to treat, improving. The statistics showed positive (89.1%), negative (1.7%), conditional (9.2%) for the polarity of the relations, and one-one (11.8%), one-many (16.7%), many-one (13.4%), many-many (12.1%) for their cardinality.
d. Examples of terms from classes [TMS] (omitted, next) & [CR-TMS-effects] & [mental disabilities] co-occurring in the same sentence (MAPP; 1290 sentences; | = OR):
  – [treat] & [depression (24%)| disorder (12%)| tinnitus (4.3%)| schizophrenia (3%)| stress (1%)| pain (1%)| stroke (1%)| epilepsy (1%)| anxiety (1%)| seizures (0.2%)| neglect (0.1%)];
  – [therapy] & [depression (5%)| stroke (3%)| pain (1%)| epilepsy (0.4%)| seizures (0.5%)| schizophrenia (0.2%)| mood (0.2%)];
  – [improve] & [stroke (3%)| depression (0.6%)| tinnitus (0.3%)| reading (0.1%)| attention (0.1%)| schizophrenia (0.1%)];
e. Relationship Extraction for CE-TMS (ANote2; 7404 annotations). From 2025 relations, 903 were verbs associated relations, and we found among the top 30 the following verbs: is, induced, applied, guided, learning, improved, increasing, enhancing, was applied, paired, stimulated, delivered, reduced, impaired, performed, encoding. The statistics showed positive (92.0%), negative (1.2%), conditional (6.8%) for the polarity of the relations, and one-one (14.0%), one-many (17.6%), many-one (14.5%), many-many (12.4%) for their cardinality.
f. Example of terms from classes [TMS] (omitted, next) & [CE-TMS-effects] & [cognitive processes] occurring in the same sentence (MAPP; 1591 sentences):
  – [performance] & [memory (3.7%)| attention (1%)| language (1%)| motor (1%)| visual (1%)];
  – [facilitation] & [motor (6%)| memory (2%)| linguistic (2%)| concept (1%)| control (1%)| knowledge (1%)| logical (1%)| awareness (1%)| visual (1%)];
  – [improve] & [memory (1%)| visual (1%)| language (1%)];
g. Example of high probability sentences (PP, range [4, 99]) from the CR-TMS corpus including terms from classes [TMS] & [CR-TMS-effects] & [mental disabilities] (see d):
  – “Daily rTMS improves mood in depression” (PP 13.0).
  – “Excitatory rTMS induces improvements in chronic post-stroke aphasia” (PP 13.5).
  – “Slow TMS can rapidly reduce resistant auditory hallucinations in schizophrenia” (PP 16.8).

Using VisualText we showed also that from 4085 sentences, 1417 include terms from [TMS] hierarchy, and 847 include terms from [TMS] & [CR-TMS-effects OR mental disabilities]. Examples from the last group:

– “TMS has been shown to be an effective treatment for mental illnesses including major depressive disorder.”
– “…rTMS has been developed for the treatment of major depression and schizophrenia.”

h. Example of high probability sentences (PP; range [3, 50]) from the CE-TMS corpus including terms from classes [TMS] & [CE-TMS-effects] & [cognitive processes] (see f):
  – “…rTMS to left dorsal premotor cortex enhances motor consolidation of new skills” (PP 9).
  – “rTMS over Wernicke's area leads to a brief facilitation of picture naming by shortening linguistic processing time” (PP 11).
  – “…rTMS at alpha frequency can modulate short-term memory capacity by influencing the ability to suppress distracting information” (PP 15).

Using VisualText we showed that from 1008 sentences, 445 included terms from [TMS], and 238 include terms from [TMS] & [CE-TMS-effects OR cognitive processes]. Examples from the last group:

– “…rTMS of the DLPFC can affect the performance in an affective go-no-go task.”
– “…here we report the ipsilateral enhancement of visual attention after rTMS of parietal cortex at parameters known to reduce cortical excitability.”

k. Example of sentences extracted with BioRAT from the CR-TMS corpus using a specific rule, which indicate classes of terms that have to be found at specific locations (block) in the sentence (Table 5).

**Table 5:** **Example sentences retrieved with BioRAT using a specific rule**.

**Blocks of the rule**						**Context**
**<LOOKUP: TMS>**	**<MACRO: WORD>**	**<MACRO: WORD?>**	**<LOOKUP: CR-TMS-effects>**	**<MACRO: WORD>**	**<LOOKUP: mental disabilities>**	
rTMS	for	–	treatment	of	depression	“… the first cases report of using rTMS for the treatment of depression … ”
TMS	for	–	treatment	of	obsessive compulsive disorder	“… TMS for the treatment of obsessive compulsive disorder … ”

*“< >” delimitate a block of the rule; LOOKUP, looking for a term included in a class ([TMS], [CR-TMS-effects] or [mental disabilities]); MACRO WORD, any word; “?”, the block is optional*.

## Discussions and conclusions

We here used a set of selected TM-tools to obtain basic insights into the relevant literature on the CR- and CE-TMS. For obvious reasons, we limited this application to few simple aspects. First, we showed that TM could retrieve from vast corpora of publications the diversity of TMS applications in CR and CE, automatically extracting trends already described in published reviews. Second, we searched for trends noticeable only in big corpora of publications.

Along this exercise, we attempted to validate our results in different ways. For example, we compared similar results obtained using different TM-tools applied to different corpora (TIAB-corpora or TIABREV-corpora). Relevant and common aspects, synthesized in unique results per type of analysis and topics were shown in the paper. Finally, we compared TM to human curation efforts. Accordingly, we selected for human curation 30 of the top ranked (with Medline Ranker) publications from the TIABREV-corpora, separately for CR-TMS and CR-TMS. We performed a selective manual curation aimed to retrieve relevant terms co-occurring with TMS (all types of protocol) in the abstracts, which belong to the following classes: mental functions, healthy or impaired, modulated by TMS; mental disabilities treated with TMS; rehabilitation or enhancement effects of TMS. We also searched for relationships between classes of relevant terms and conclusive sentences summarizing research results.

Very briefly, the manual curation gave the following perspective over the main topics:

- CR-TMS. TMS is continuously establishing itself as one of the “tools of the trade” in psychiatric therapeutic practice (Kammer and Spitzer, 2012) improving mental functions in: Parkinson's disease (Pascual-Leone et al., 1994), aphasia (Medina et al., 2012), motor control after stroke (Takeuchi et al., 2005), epilepsy (Nitsche and Paulus, 2009), depression (Lisanby et al., 2009; Conforto et al., 2014), schizophrenia (Levkovitz et al., 2011; Kammer and Spitzer, 2012), autism (Krause et al., 2012), chronic migraine (Conforto et al., 2014), dyslexia (Costanzo et al., 2013), neglect (Fasotti and Van Kessel, 2013), obsessive-compulsive disorder (OCD) (Mantovani et al., 2013), chronic pain (Moreno-Duarte et al., 2014), and social anxiety disorder (Paes et al., 2013). The TMS therapy applied to younger patients (children and adolescents) improves cognitive functions (Vicario and Nitsche, 2013) in: stroke affecting the motor cortex (Kirton et al., 2008), epilepsy (Fregni et al., 2005), ADHD (Weaver et al., 2012), Tourette syndrome (Le et al., 2013), autism (Baruth et al., 2010), treatment-resistant depression (Bloch et al., 2008), and medication-resistant schizophrenia (Jardri et al., 2012).
- CE-TMS. CE is defined as any augmentation of core information processing systems in the brain underlying perception, attention, conceptualization, memory, reasoning and motor performance (Sandberg and Bostrom, 2006; Luber and Lisanby, 2014). Studies reported TMS-induced modulations and enhancements of brain functioning and neural processing involved in: language comprehension (Floel et al., 2008), learning and memory (Vicario et al., 2013), cortical plasticity improving learning (Vallence and Ridding, 2014), motor memory (Butefisch et al., 2004), working memory (Gaudeau-Bosma et al., 2013), memory (Gagnon et al., 2011; Blumenfeld et al., 2014), phonological memory (Kirschen et al., 2006), perception (Hamilton et al., 2013), perceptual discrimination (Luber and Lisanby, 2014), eye movements and visual search, (Gerits et al., 2011; Luber and Lisanby, 2014), attention (Cooper et al., 2004; Lee et al., 2013), reward behavior (Stanford et al., 2013), analogic reasoning (Boroojerdi et al., 2001), motor learning (Luber and Lisanby, 2014), consolidation of new skills (Boyd and Linsdell, 2009), visual awareness (Grosbras and Paus, 2003), activity of specific frequencies supporting functions of the brain (Rahnev, 2013), and Pavlovian conditioning (Luber et al., 2007).

CR-TMS and CE-TMS used various TMS paradigms, including single-pulse, theta-burst, paired-pulse, and trains of rTMS at both low and high frequencies (Luber and Lisanby, 2014).

Comparisons with the manual curation showed that the TM-tools were also able to extract:

– All the relevant terms for CR-TMS and CE-TMS in the form of: lists; topics; classes of terms associated with specific subtopics (e.g., mental disabilities, cognitive processes).
– Relations between relevant terms in the form of: co-occurrences maps (Figure 3); groups of relevant terms with high probabilities co-occurrences; KWIC (Tables 3, 4); lists of relevant relational verbs.
– High probability and relevance conclusive sentences (see examples and Table 5). We studied also structural statistical regularities in both conclusive sentences and abstracts shown by: the relative position in the sentence for groups of relevant terms (Tables 3–5); combinations of relevant terms with high probability occurrence; the occurrence frequency for conclusive sentences.

In addition, the TM approach has clear advantages emerging from the statistical properties of big corpora. Accordingly, the tirade (terms, terms-relationships, sentences) gained statistical strength, enabling us to quantify the frequency of a term or occurrence probabilities for specific relationships between terms or for conclusive sentences. For example, the hierarchy of the top terms for the CR-TMS-corpus includes TMS, treatment, rTMS, brain, therapy, depression, and stroke. We also found hierarchies for classes of terms like [CR-TMS-effects] (e.g., treatment, therapy, recovery, antidepressant, rehabilitation, neurorehabilitation) and [mental disabilities] (e.g., depression, stroke, schizophrenia, tinnitus, major depressive disorder, Parkinson, OCD, epilepsy, seizures, neglect, anxiety, ADHD, stress, Alzheimer's). For the CE-TMS-corpus the top terms are TMS, rTMS, brain, cognitive, performance, and facilitation. We also added hierarchies for classes of terms like [CE-TMS-effects] (e.g., performance enhancement, improvement, facilitation, neuromodulation, neurostimulation, therapy, neuroenhancement, rehabilitation, CE) and [cognitive processes] (e.g., memory, learning, attention, working memory, perception, language skill acquisition, decision, emotion, speech, semantic processing).

The relevance of all the retrieved terms and of their relationships is based on the idea that words co-occurring frequently in the abstracts are related in specific ways intrinsically constrained by the (TMS-related) topic of the abstracts. Thus, TMS is strongly related with all frequent terms retrieved with different TM-tools. Although in a relatively crude form, determined by our intention to show “raw” TM results, our study is showing that TMS emerged as one of the important non-invasive tools that can both improve cognitive and motor functions in numerous neurological diseases and induce enhancements of many fundamental brain functions.

We were able to characterize topics considering their dynamic relationships, trends in research and the interest shown by the scientific community. For example, CR-TMS and CE-TMS share studies (Figure 4), being an argument for their similarity. The reviewed topics share also publications with other fields suggesting their appurtenance to a larger context, which integrates diagnostic, fundamental research and fMRI studies. TMS can be used both to investigate and to modify brain physiology and performance in healthy and diseased subjects (Vicario and Nitsche, 2013).

Methodologically speaking, we conclude that TM was helpful in getting an overall perspective on a huge corpus of literature with some level of detail, intentionally limited to handle complexity. Richer information can be extracted using more complex TM methods focused on narrower topics, but this requires extensive training and knowledge.

A decision factor to use TM relates to how profitable and how difficult the tools may be. The study aimed to address these simple issues in a pragmatic way. First and foremost, we argue that TM-tools may become a basic component in the methodological library. Unfortunately, it is equally clear that TM is a difficult task. With this in mind, we aimed to evaluate relatively immediate advantages of a user-friendly TM approach, based on easy-to-use TM-tools applied to CR- and CE-TMS corpora of abstracts. The hierarchical structure of our example set of TM-tools could serve as a guide for researchers aiming to use TM. Accordingly, for a rapid enrichment of the PubMed search, TM-tools II could be used, with special considerations for Carrot2, PubReMiner, Quertle, Medline Ranker, and Textpresso for Neuroscience. All TM-tools III could help a more elaborate TM without a considerable increase in demands to the user. For complex studies combining multiple aspects of the “mining,” we recommend systems like Knime, RapidMiner, and Taverna.

### Conflict of interest statement

The authors declare that the research was conducted in the absence of any commercial or financial relationships that could be construed as a potential conflict of interest.

## References

[B1] Akil H. Martone M. E. Van Essen D. C. (2011). Challenges and opportunities in mining neuroscience data. Science 331, 708–712 10.1126/science.1199305 21311009 PMC3102049

[B2] Alfred R. Razali M. Alias S. On C. (2014). A visualization approach to automatic text documents categorization based on HAC, in The 8th International Conference on Knowledge Management in Organizations, Social and Big Data Computing for Knowledge Management, KMO 2013, eds Uden L. Wang L. S. L. Corchado Rodríguez J. M. Yang H.-C. Ting I. H. (Kaohsiung: Springer), 273–284 10.1007/978-94-007-7287-8_22

[B3] Baruth J. M. Casanova M. F. El-Baz A. Horrell T. Mathai G. Sears L. (2010). Low-frequency repetitive transcranial magnetic stimulation (rTMS) modulates evoked-gamma frequency oscillations in autism spectrum disorder (ASD). J. Neurother. 14, 179–194 10.1080/10874208.2010.501500 21116441 PMC2992386

[B4] Becker K. G. Hosack D. A. Dennis G. Jr. Lempicki R. A. Bright T. J. Cheadle C. (2003). PubMatrix: a tool for multiplex literature mining. BMC Bioinform. 4:61 10.1186/1471-2105-4-61 14667255 PMC317283

[B5] Bloch Y. Grisaru N. Harel E. V. Beitler G. Faivel N. Ratzoni G. (2008). Repetitive transcranial magnetic stimulation in the treatment of depression in adolescents: an open-label study. J. ECT 24, 156–159 10.1097/YCT.0b013e318156aa49 18580562

[B6] Blumenfeld R. S. Lee T. G. D'Esposito M. (2014). The effects of lateral prefrontal transcranial magnetic stimulation on item memory encoding. Neuropsychologia 53, 197–202 10.1016/j.neuropsychologia.2013.11.021 24316198 PMC4394733

[B7] Boroojerdi B. Phipps M. Kopylev L. Wharton C. M. Cohen L. G. Grafman J. (2001). Enhancing analogic reasoning with rTMS over the left prefrontal cortex. Neurology 56, 526–528 10.1212/WNL.56.4.526 11222799

[B8] Boyd L. A. Linsdell M. A. (2009). Excitatory repetitive transcranial magnetic stimulation to left dorsal premotor cortex enhances motor consolidation of new skills. BMC Neurosci. 10:72 10.1186/1471-2202-10-72 19583831 PMC2713248

[B9] Brem A. K. Fried P. J. Horvath J. C. Robertson E. M. Pascual-Leone A. (2014). Is neuroenhancement by noninvasive brain stimulation a net zero-sum proposition? Neuroimage 85 Pt 3, 1058–1068 10.1016/j.neuroimage.2013.07.038 23880500 PMC4392930

[B10] Bridges-Webb C. (1986). A computer summary for general practice medical records: MEDSUM. J. Fam. Pract. 23, 389–392 3760805

[B11] Butefisch C. M. Khurana V. Kopylev L. Cohen L. G. (2004). Enhancing encoding of a motor memory in the primary motor cortex by cortical stimulation. J. Neurophysiol. 91, 2110–2116 10.1152/jn.01038.2003 14711974

[B12] Carpineto C. Osinski S. Romano G. Weiss D. (2009). A survey of Web clustering engines. ACM Comput. Surv. 41, 1–38 10.1145/1541880.154188417911696

[B13] Cohen K. B. Hunter L. (2008). Getting started in text mining. PLoS Comput. Biol. 4:e20 10.1371/journal.pcbi.0040020 18225946 PMC2217579

[B14] Conforto A. B. Amaro E. Jr. Goncalves A. L. Mercante J. P. Guendler V. Z. Ferreira J. R. (2014). Randomized, proof-of-principle clinical trial of active transcranial magnetic stimulation in chronic migraine. Cephalalgia 34, 464–472 10.1177/0333102413515340 24326236

[B15] Cooper A. C. Humphreys G. W. Hulleman J. Praamstra P. Georgeson M. (2004). Transcranial magnetic stimulation to right parietal cortex modifies the attentional blink. Exp. Brain Res. 155, 24–29 10.1007/s00221-003-1697-9 15064881

[B16] Corney D. P. Buxton B. F. Langdon W. B. Jones D. T. (2004). BioRAT: extracting biological information from full-length papers. Bioinformatics 20, 3206–3213 10.1093/bioinformatics/bth386 15231534

[B17] Costanzo F. Menghini D. Caltagirone C. Oliveri M. Vicari S. (2013). How to improve reading skills in dyslexics: the effect of high frequency rTMS. Neuropsychologia 51, 2953–2959 10.1016/j.neuropsychologia.2013.10.018 24184439

[B18] Dias A. M. Mansur C. G. Myczkowski M. Marcolin M. (2011). Whole field tendencies in transcranial magnetic stimulation: a systematic review with data and text mining. Asian J. Psychiatr. 4, 107–112 10.1016/j.ajp.2011.03.003 23051076

[B19] Doms A. Schroeder M. (2005). GoPubMed: exploring PubMed with the Gene Ontology. Nucleic Acids Res. 33, W783–W786 10.1093/nar/gki470 15980585 PMC1160231

[B20] Fasotti L. Van Kessel M. (2013). Novel insights in the rehabilitation of neglect. Front. Hum. Neurosci. 7:780 10.3389/fnhum.2013.00780 24298249 PMC3828556

[B21] Floel A. Rosser N. Michka O. Knecht S. Breitenstein C. (2008). Noninvasive brain stimulation improves language learning. J. Cogn. Neurosci. 20, 1415–1422 10.1162/jocn.2008.20098 18303984

[B22] Fontaine J.-F. Barbosa-Silva A. Schaefer M. Huska M. R. Muro E. M. Andrade-Navarro M. A. (2009). MedlineRanker: flexible ranking of biomedical literature. Nucleic Acids Res. 37, W141–W146 10.1093/nar/gkp353 19429696 PMC2703945

[B23] Fontelo P. Liu F. Ackerman M. (2005). askMEDLINE: a free-text, natural language query tool for MEDLINE/PubMed. BMC Med. Inform. Decis. Mak. 5:5 10.1186/1472-6947-5-5 15760470 PMC1079856

[B24] Fregni F. Thome-Souza S. Bermpohl F. Marcolin M. A. Herzog A. Pascual-Leone A. (2005). Antiepileptic effects of repetitive transcranial magnetic stimulation in patients with cortical malformations: an EEG and clinical study. Stereotact. Funct. Neurosurg. 83, 57–62 10.1159/000086674 15990468

[B25] Gagnon G. Schneider C. Grondin S. Blanchet S. (2011). Enhancement of episodic memory in young and healthy adults: a paired-pulse TMS study on encoding and retrieval performance. Neurosci. Lett. 488, 138–142 10.1016/j.neulet.2010.11.016 21094215

[B26] Gaudeau-Bosma C. Moulier V. Allard A. C. Sidhoumi D. Bouaziz N. Braha S. (2013). Effect of two weeks of rTMS on brain activity in healthy subjects during an n-back task: a randomized double blind study. Brain Stimul. 6, 569–575 10.1016/j.brs.2012.10.009 23194830

[B27] Gerits A. Ruff C. Guipponi O. Wenderoth N. Driver J. Vanduffel W. (2011). Transcranial magnetic stimulation of macaque frontal eye fields decreases saccadic reaction time. Exp. Brain Res. 212, 143–152 10.1007/s00221-011-2710-3 21544509

[B28] Giglia E. (2011). Quertle and KNALIJ: searching PubMed has never been so easy and effective. Eur. J. Phys. Rehabil. Med. 47, 687–690 22222966

[B29] Grosbras M. H. Paus T. (2003). Transcranial magnetic stimulation of the human frontal eye field facilitates visual awareness. Eur. J. Neurosci. 18, 3121–3126 10.1111/j.1460-9568.2003.03055.x 14656308

[B30] Hamilton R. H. Wiener M. Drebing D. E. Coslett H. B. (2013). Gone in a flash: manipulation of audiovisual temporal integration using transcranial magnetic stimulation. Front. Psychol. 4:571 10.3389/fpsyg.2013.00571 24062701 PMC3769638

[B31] Higuchi K. (2012). The internet in newspaper articles and people's minds: a corpus-based exploratory approach to social consciousness in japan, in The 4th International Conference on Corpus (Linguistics), 115–116

[B32] Hoskinson A. (2005). Creating the ultimate research assistant. Computer 38, 97–99 10.1109/MC.2005.375

[B33] Iliopoulos I. Enright A. J. Ouzounis C. A. (2001). Textquest: document clustering of Medline abstracts for concept discovery in molecular biology. Biocomputing 2001, 384–395 10.1142/9789814447362_0038 11262957

[B34] Jardri R. Bubrovszky M. Demeulemeester M. Poulet E. Januel D. Cohen D. (2012). Repetitive transcranial magnetic stimulation to treat early-onset auditory hallucinations. J. Am. Acad. Child Adolesc. Psychiatry 51, 947–949 10.1016/j.jaac.2012.06.010 22917208

[B35] Kammer T. Spitzer M. (2012). Brain stimulation in psychiatry: methods and magnets, patients and parameters. Curr. Opin. Psychiatry 25, 535–541 10.1097/YCO.0b013e328358df8c 22992545

[B36] Kirschen M. P. Davis-Ratner M. S. Jerde T. E. Schraedley-Desmond P. Desmond J. E. (2006). Enhancement of phonological memory following transcranial magnetic stimulation (TMS). Behav. Neurol. 17, 187–194 10.1155/2006/469132 17148839 PMC5471529

[B37] Kirton A. Chen R. Friefeld S. Gunraj C. Pontigon A. M. Deveber G. (2008). Contralesional repetitive transcranial magnetic stimulation for chronic hemiparesis in subcortical paediatric stroke: a randomised trial. Lancet Neurol. 7, 507–513 10.1016/S1474-4422(08)70096-6 18455961

[B38] Koster J. (2008). PubMed. PubReMiner. Available online at: http://hgserver2.amc.nl/cgi-bin/miner/miner2.cgi

[B39] Krause L. Enticott P. G. Zangen A. Fitzgerald P. B. (2012). The role of medial prefrontal cortex in theory of mind: a deep rTMS study. Behav. Brain Res. 228, 87–90 10.1016/j.bbr.2011.11.037 22155478

[B40] Landauer T. K. Foltz P. W. Laham D. (1998). An introduction to latent semantic analysis. Discourse Process. 25, 259–284 10.1080/01638539809545028

[B41] Le K. Liu L. Sun M. Hu L. Xiao N. (2013). Transcranial magnetic stimulation at 1 Hertz improves clinical symptoms in children with Tourette syndrome for at least 6 months. J. Clin. Neurosci. 20, 257–262 10.1016/j.jocn.2012.01.049 23238046

[B42] Lee J. Ku J. Han K. Park J. Lee H. Kim K. R. (2013). rTMS over bilateral inferior parietal cortex induces decrement of spatial sustained attention. Front. Hum. Neurosci. 7:26 10.3389/fnhum.2013.00026 23403477 PMC3568694

[B43] Levkovitz Y. Rabany L. Harel E. V. Zangen A. (2011). Deep transcranial magnetic stimulation add-on for treatment of negative symptoms and cognitive deficits of schizophrenia: a feasibility study. Int. J. Neuropsychopharmacol. 14, 991–996 10.1017/S1461145711000642 21524336

[B44] Lisanby S. Husain M. Rosenquist P. Maixner D. Gutierrez R. Krystal A. (2009). Daily left prefrontal repetitive transcranial magnetic stimulation in the acute treatment of major depression: clinical predictors of outcome in a multisite, randomized controlled clinical trial. Neuropsychopharmacology 34, 522–534 10.1038/npp.2008.118 18704101

[B45] Lourenco A. Carreira R. Carneiro S. Maia P. Glez-Pena D. Fdez-Riverola F. (2009). @Note: a workbench for biomedical text mining. J. Biomed. Inform. 42, 710–720 10.1016/j.jbi.2009.04.002 19393341

[B46] Lu Z. (2011). PubMed and beyond: a survey of web tools for searching biomedical literature. Database (Oxford) 2011:baq036 10.1093/database/baq036 21245076 PMC3025693

[B47] Luber B. Balsam P. Nguyen T. Gross M. Lisanby S. H. (2007). Classical conditioned learning using transcranial magnetic stimulation. Exp. Brain Res. 183, 361–369 10.1007/s00221-007-1052-7 17639360

[B48] Luber B. Lisanby S. H. (2014). Enhancement of human cognitive performance using transcranial magnetic stimulation (TMS). Neuroimage 85(Pt 3), 961–970 10.1016/j.neuroimage.2013.06.007 23770409 PMC4083569

[B49] Mantovani A. Rossi S. Bassi B. D. Simpson H. B. Fallon B. A. Lisanby S. H. (2013). Modulation of motor cortex excitability in obsessive-compulsive disorder: an exploratory study on the relations of neurophysiology measures with clinical outcome. Psychiatry Res. 210, 1026–1032 10.1016/j.psychres.2013.08.054 24064461 PMC7325264

[B50] McCallum A. K. (2002). MALLET: A Machine Learning for Language Toolkit. Available online at: http://mallet.cs.umass.edu

[B51] Medina J. Norise C. Faseyitan O. Coslett H. B. Turkeltaub P. E. Hamilton R. H. (2012). Finding the right words: transcranial magnetic stimulation improves discourse productivity in non-fluent aphasia after stroke. Aphasiology 26, 1153–1168 10.1080/02687038.2012.710316 23280015 PMC3532848

[B52] Meyers A. (2003). Multi-Pass Multi-Strategy Nlp. Available online at: http://www.textanalysis.com/tai-multi2003.pdf

[B53] Miniussi C. Rossini P. M. (2011). Transcranial magnetic stimulation in cognitive rehabilitation. Neuropsychol. Rehabil. 21, 579–601 10.1080/09602011.2011.562689 21462081

[B54] Moreno-Duarte I. Morse L. R. Alam M. Bikson M. Zafonte R. Fregni F. (2014). Targeted therapies using electrical and magnetic neural stimulation for the treatment of chronic pain in spinal cord injury. Neuroimage 85 Pt 3, 1003–1013 10.1016/j.neuroimage.2013.05.097 23727533

[B55] Muller H. M. Rangarajan A. Teal T. K. Sternberg P. W. (2008). Textpresso for neuroscience: searching the full text of thousands of neuroscience research papers. Neuroinformatics 6, 195–204 10.1007/s12021-008-9031-0 18949581 PMC2666735

[B56] Nitsche M. A. Paulus W. (2009). Noninvasive brain stimulation protocols in the treatment of epilepsy: current state and perspectives. Neurotherapeutics 6, 244–250 10.1016/j.nurt.2009.01.003 19332316 PMC5084200

[B57] Paes F. Baczynski T. Novaes F. Marinho T. Arias-Carrion O. Budde H. (2013). Repetitive transcranial magnetic stimulation (rTMS) to treat social anxiety disorder: case reports and a review of the literature. Clin. Pract. Epidemiol. Ment. Health 9, 180–188 10.2174/1745017901309010180 24278088 PMC3837365

[B58] Pascual-Leone A. Valls-Sole J. Brasil-Neto J. P. Cammarota A. Grafman J. Hallett M. (1994). Akinesia in Parkinson's disease. II. Effects of subthreshold repetitive transcranial motor cortex stimulation. Neurology 44, 892–898 10.1212/WNL.44.5.892 8190293

[B59] Perez-Iratxeta C. Bork P. Andrade M. A. (2001). XplorMed: a tool for exploring MEDLINE abstracts. Trends Biochem. Sci. 26, 573–575 10.1016/S0968-0004(01)01926-0 11551795

[B60] Rahnev D. (2013). Entrainment of neural activity using transcranial magnetic stimulation. J. Neurosci. 33, 11325–11326 10.1523/JNEUROSCI.2012-13.2013 23843505 PMC6618692

[B61] Sandberg A. Bostrom N. (2006). Converging cognitive enhancements. Ann. N.Y. Acad. Sci. 1093, 201–227 10.1196/annals.1382.015 17312260

[B62] Sarkar I. N. Schenk R. Miller H. Norton C. N. (2009). LigerCat: using “MeSH Clouds” from journal, article, or gene citations to facilitate the identification of relevant biomedical literature. AMIA Annu. Symp. Proc. 2009, 563–567 20351918 PMC2815376

[B63] Smalheiser N. R. Zhou W. Torvik V. I. (2008). Anne O'Tate: a tool to support user-driven summarization, drill-down and browsing of PubMed search results. J. Biomed. Discov. Collab. 3:2 10.1186/1747-5333-3-2 18279519 PMC2276193

[B64] Stanford A. D. Luber B. Unger L. Cycowicz Y. M. Malaspina D. Lisanby S. H. (2013). Single pulse TMS differentially modulates reward behavior. Neuropsychologia 51, 3041–3047 10.1016/j.neuropsychologia.2013.09.016 24041669

[B65] Takeuchi N. Chuma T. Matsuo Y. Watanabe I. Ikoma K. (2005). Repetitive transcranial magnetic stimulation of contralesional primary motor cortex improves hand function after stroke. Stroke 36, 2681–2686 10.1161/01.STR.0000189658.51972.34 16254224

[B66] Vallence A. M. Ridding M. C. (2014). Non-invasive induction of plasticity in the human cortex: uses and limitations. Cortex 58C, 261–271 10.1016/j.cortex.2013.12.006 24439754

[B67] Vicario C. M. Candidi M. Aglioti S. M. (2013). Cortico-spinal embodiment of newly acquired, action-related semantic associations. Brain Stimul. 6, 952–958 10.1016/j.brs.2013.05.010 23856556

[B68] Vicario C. M. Nitsche M. A. (2013). Non-invasive brain stimulation for the treatment of brain diseases in childhood and adolescence: state of the art, current limits and future challenges. Front. Syst. Neurosci. 7:94 10.3389/fnsys.2013.00094 24324410 PMC3838957

[B69] Weaver L. Rostain A. L. Mace W. Akhtar U. Moss E. O'Reardon J. P. (2012). Transcranial magnetic stimulation (TMS) in the treatment of attention-deficit/hyperactivity disorder in adolescents and young adults: a pilot study. J. ECT 28, 98–103 10.1097/YCT.0b013e31824532c8 22551775

[B70] Zeimpekis D. Gallopoulos E. (2006). TMG: a MATLAB toolbox for generating term-document matrices from text collections, in Grouping Multidimensional Data: Recent Advances in Clustering, eds Kogan J. Nicholas C. Teboulle M. (Berlin, Heidelberg: Springer), 187–210

[B71] Zhu M. Ghodsi A. (2006). Automatic dimensionality selection from the scree plot via the use of profile likelihood. Comput. Stat. Data Anal. 51, 918–930 10.1016/j.csda.2005.09.010

